# Analysis on the literature communication path of new media integrating public mental health

**DOI:** 10.3389/fpsyg.2022.997558

**Published:** 2022-09-20

**Authors:** Shaojing Liu

**Affiliations:** ^1^School of Chinese Language and Literature, Linyi University, Linyi, China; ^2^Center for Literary Theory and Aesthetics, School of Literature, Shandong University, Jinan, China

**Keywords:** mental health, communication paths, literary works, new media, increasing pressure

## Abstract

The communication of mental health is an important branch of health communication, and it is also an important factor affecting people’s physical and mental health. With the increasing pressure of life, people’s mental health problems have huge challenges. Under the enormous pressure of economy and life, people’s mental health problems are becoming increasingly prominent. This calls for attention to mental health issues. In the context of new media, knowledge about mental health can be disseminated through the Internet and mobile platforms. This approach will spread awareness of mental health prevention and basic issues. Mental health problems are also a manifestation of the lack of humanistic spirit. Excellent works related to humanistic spirit can promote the relief of mental health problems. Literature can contribute to the development of mental health problems. This research studies the communication of mental health issues in the context of new media using literary works as a carrier. At the same time, it also considers big data-related algorithms to mine the traditional characteristics of mental health problems. The research results show that new media technology can well assist the dissemination of mental health education, and literary works also contribute to the dissemination of mental health education knowledge. Collaborative filtering algorithm and atrous convolution algorithm can better predict the relevant characteristics in the process of mental health communication. For the *CF* algorithm, its maximum similarity index reached 0.987 when recommending mental health propagation paths using new media technology. For ACNN, the smallest prediction error is only 1.78%.

## Introduction

Mental health has always been an important health issue, and it is easy to overlook. Because mental health problems are different from other types of physical health problems (Hassan et al., 2018; Kotera and Maughan, 2020). In the early days, life was less stressful and economic development was slower, which made it harder to cause serious mental health problems. That is why mental health issues are so hard to get people’s attention. However, with the increasing pressure of life and the rapid economic development, this can easily lead to mental health problems. The imbalance between the improvement of living standards and economic income is also the main cause of mental health problems (Hsiung et al., 2019; Doblyte, 2020). The lack of humanistic spirit is also the cause of people’s mental health problems. Mental health problems are a relatively hidden physical problem, and it is not easy to be discovered. People also reluctantly admit to their mental health problems. This also has a greater relationship with people’s perception of mental health problems. Most people have misunderstandings about mental health problems (Kotera et al., 2019; Wollast et al., 2020). Mental health problems are considered to be a less friendly health problem. However, most people are prone to mental health problems due to the influence of life or school. Students’ boredom with their studies is also a simple mental health problem, and people’s dissatisfaction with life is also a simple mental health problem. If the pressure of life or school is constantly reduced, this will prevent the further expansion of mental health problems. However, if people cannot deal with the relationship between life, study and psychology well, it will easily lead to the collapse of psychological problems, which is a serious mental health problem (Kotera et al., 2019; Mirzaei et al., 2019). Therefore, the dissemination of mental health and publicity and education is also a relatively important topic. Whether it is for life or school, if mental health problems can be promoted in a timely and correct manner, this will lead to fewer people with mental health problems in society. This will also reduce crime rates at the societal level. The main reason for the relatively large defects in the publicity of mental health problems is that people’s cognition and attention to mental health problems are not enough. And people’s publicity paths and publicity methods for mental health problems are a big problem (Sickel et al., 2019; Lee and Seo, 2020). With the development of new media technology, publicity and dissemination of mental health problems has become a possibility. New media technology can allow mental health issues to be communicated through the Internet and mobile clients. The advantage of new media technology in disseminating mental health issues is that it can well protect the privacy of beneficiaries, which is also a form of publicity that is more acceptable to the public.

At the same time, literary works can better reflect a positive humanistic spirit. The emergence of mental health problems is related to the problems of humanistic spirit. Literary works can guide people to develop toward a positive life direction. In short, literary works are a kind of works that are beneficial to people’s mental health problems (Brown, 2018; Cross and Cheyne, 2018). Changes in the social environment are closely related to the occurrence of mental health problems. Problems such as further studies, unemployment, mortgages, and emotions are all factors that lead to mental health problems. With the advent of the economic era of reform and opening up, there will be some unfairness and market competition in the social economy, which will lead to problems such as instant collapse of the economy (Hsiung et al., 2019; Adams and Walls, 2020). This can lead to irritability and poor mental health in people. People’s continuous pursuit of the quality of life will lead to the reduction of people’s pursuit of spiritual level. The improvement of the quality of life leads to the decline of the humanistic spirit level. Although new media technology can spread customs and culture, and this way is a relatively rapid and acceptable way to the public. However, the process of new media traditional culture will inevitably produce some bad cultures, and these bad contents will easily lead to a kind of comparison and radical psychology. This can also lead to more serious psychological problems (Kim and Ko, 2018; Kotera et al., 2019). Therefore, the dissemination of cultural works will also benefit mental health education (Nelson et al., 2019; Kobayashi et al., 2020). In short, the dissemination of cultural works will also be conducive to the dissemination of mental health problems. This research mainly studies the communication path of mental health problems using new media technology in combination with literary works.

This study examines the problem of transmission paths for mental health problems using new media technologies. However, there will be more features and data involved here. Cumbersome nonlinear relationships and huge amounts of data are more difficult tasks for manual means. Manually dealing with the problem of mental health transmission paths will not only consume a lot of time, but also consume a lot of material resources. Manual methods also have a higher error rate. This study considers the application of big data technology to the problems of mental health and literary communication. Big data technology has been successfully proven to be a technology that is good at dealing with tedious data (Tetila et al., 2019; Qiu et al., 2022). Big data technology includes many kinds of feature extraction algorithms. In this study, considering the relationship between mental health and literature communication, an atrous convolutional neural network (ACNN) algorithm was used to extract communication-related feature data (Tan et al., 2018; Fu et al., 2019). In order to facilitate the identification rate of the propagation paths of mental health problems, collaborative filtering algorithms were also considered in this study. The collaborative filtering algorithm can realize the active recommendation of research objects, and it can realize the recommendation of related features and data by calculating the distance between different data (Lu et al., 2019; Tian, 2021). The ACNN method is a special convolution operation method, which can also perform feature extraction. However, the ACNN method can be trained and iterated more efficiently. The *CF* algorithm can complete the active recommendation function of the new media propagation path.

This study analyzes the relationship between mental health and literary dissemination paths using new media technologies. New media technology can be used as a path for mental health and literature dissemination, and this method is also a new method of mental health-related knowledge dissemination. There is a similar relationship between literary works and mental health problems. However, a reasonable dissemination path will also affect the effect of the dissemination of mental health knowledge. This research mainly uses 5 sections to analyze the influence and effect of mental health and literary knowledge in the process of new media communication. Section 1 presents the significance of mental health issues and the relationship of literature to mental health issues. Section 2 investigates the current state of research on mental health issues. Research proposals on mental health and literary communication pathways are analyzed in Section 3 through new media technologies. Section 4 analyzes the application feasibility of ACNN and *CF* intelligent algorithms in mental health and literature dissemination paths. This is also a key content of the study, and it is also a quantitative analysis method to study the propagation path. This study uses statistical parameters such as mean error, mental health feature prediction scatterplot, and mental health-related feature prediction box plot to describe the accuracy of prediction. Section 5 summarizes the dissemination paths of mental health and the application of new media technologies.

## Related work

Mental health problems are facing a serious test, it is not only harmful to the individual, it also has a negative impact on society. This is mainly caused by the pressure of life and studies, and it also has an important relationship with the lack of humanistic spirit. The publicity and dissemination of mental health education is particularly important in today’s era. In the context of today’s environment, many researchers have done a lot of research on mental health education. Kotera et al. (2021) has also found that the psychological problems of college students have seriously affected the development and construction of a country. It mainly studies the mental health problems of Malaysian college students. It mainly studies the mental health problems of Malaysian students, the problems caused by negative mental health problems. It uses the t-test method to carry out a correlation analysis on students’ mental health problems, and it also studies the adjustment methods of mental health problems. The findings suggest that mental health problems are positively related to attitudes toward mental health. This research has specific and important implications for the recovery of students with mental health problems. Docrat et al. (2019) mainly analyzes mental health problems in low-income and middle-developing countries, and the research objects are mainly concentrated in South Africa. The lack of mental health data has led to a lag in research on mental health issues. It uses the form of a survey questionnaire to study the proportion and importance of state spending on public mental health issues. This study is the first to examine nationally representative data on mental health issues, and it also illustrates the inefficiencies of the state and the absence of other constraints on mental health issues. The research contributes to national research on mental health issues. Jun and Choi (2020) uses relevant research indicators to evaluate the relationship between mental health problems and community integration. It believes that the promotion of community festivals will reduce the incidence of mental health problems. The subjects of this study were mental health recovery persons in a Korean community. It also examines the relationship between mental health and community development using hierarchical regression analysis. The results show that this method has high accuracy. Arnot et al. (2021) argues that an individual’s experiences and attitudes toward life can affect an individual’s mental health, which in turn affects their perception of the problems they encounter, which in turn affects mental health education issues. It also found that the public is less aware of mental health knowledge and education. It looked at public mental health issues using a large UK sample. It mainly analyzes the causal relationship of mental health, the impact of mental health on the body, and the variability of mental health. The results of the study found that public mental health issues have an important role in future quantitative analysis. This kind of research method can also better transmit the related knowledge of mental health to the public. Guntzviller et al. (2020) has found that the national health system will include the assessment and improvement of mental health, which illustrates the importance of mental health in the national health system. It also surveys mental health issues in the American population in the form of a questionnaire. The findings suggest an inverse relationship between stress and communication in people with mental health problems. There was also a large negative correlation between perceived support and stress. The research has specific implications for improving people’s perceptions of mental health issues. Liang et al. (2022) mainly explores issues related to children’s mental health education. It uses big data and artificial intelligence methods to study the impact of mental health education on children and college students. It uses the Internet of Things technology and the method of convolutional neural network to build a platform for mental health education research. The results show that CNN has better performance in studying the mental health problems of children and college students. This research also has high value in studying the dissemination of mental health. This study uses the *CF* algorithm to realize the recommendation function of mental health and literature communication paths, and it also combines new media technology. This method is more innovative compared to the existing research status.

### The application of new media and big data technology in the dissemination of mental health literature

#### The importance of new media technology and big data technology

This research mainly explores the communication path of mental health integration literature communication, which applies big data technology and new media technology (He et al., 2019). In today’s era, it is an era of developed networks. New media technologies have demonstrated powerful capabilities in video as well as audio communication. However, every user will have a certain preference for new media. In order to achieve the optimal path for the dissemination of mental health integrated literature, it is not only necessary to find a suitable new media dissemination path, but also to find more people who need mental health needs (Ben-Zeev et al., 2018; Torous et al., 2020). Mental health has become a humanistic spirit, and it needs to be used by people who pay more attention to their own mental health. Therefore, this study needs to make recommendations based on the mental health-related characteristics learned by big data technology (Bojorquez et al., 2018; Carpenter-Song et al., 2020). This is a more efficient and accurate way.

#### Scheme design of the research on the communication path of mental health integration literature

**Figure 1:**
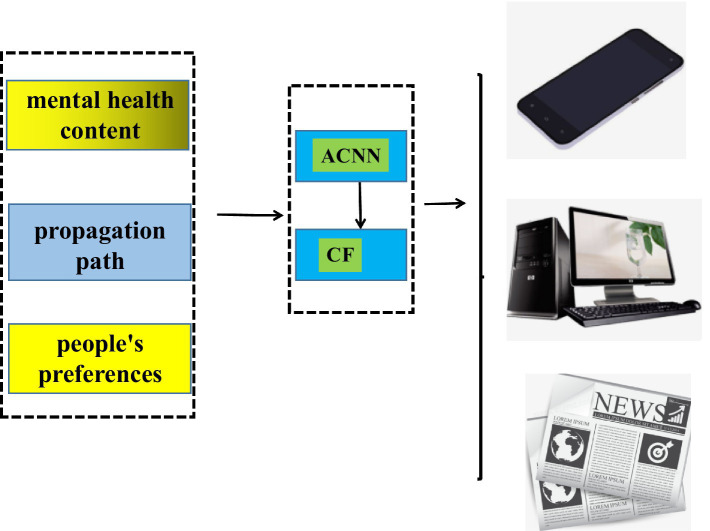
Design of communication programs for mental health literature through ACNN and new media mediums.

The dissemination path of public mental health integration literature is the key to the success of publicizing mental health-related knowledge. Various types of new media communication media have appeared in life, which can be transmitted in the form of video or audio and text, which needs to be based on the needs and hobbies of different groups of people. Therefore, the dissemination path of public mental health integration literature is particularly important. With the help of new media and the atrous convolution algorithm and collaborative filtering algorithm in big data technology, this study studies the relevant characteristics and propagation paths of public mental health fusion literature. Figure 1 shows the application process of new media and big data technology in the mental health communication path scheme. In this study, the gradient descent method is used to train the ACNN method, and the gradient descent method will find the optimal weight distribution of the characteristics related to the transmission path of mental health. It is written in Python language. First, it will capture mental health and literature-related text, audio and video features from different new media, which is the basis of big data technology learning. Then, these characteristics will be divided into three types of characteristics, which include the content characteristics of mental health, the characteristics of communication media and the characteristics of the needs of the population. These three features will go through ACNN and *CF* algorithms. Finally, the *CF* algorithm will recommend the optimal dissemination paths for different mental health literature content. In Figure 1, after the values of mental health and literature-related characteristics are passed through ACNN and *CF* algorithms, it will be recommended to relevant new media, which mainly include mobile phones, computers, and newspapers.

#### Introduction and formulas of collaborative filtering algorithms

**Figure 2:**
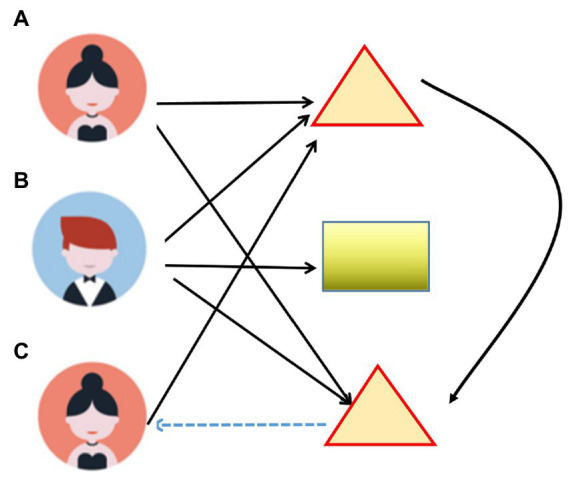
Application of *CF* algorithm in mental health transmission path.

In today’s era, e-commerce business has achieved great success, which also benefits from the recommendation function of collaborative filtering algorithm. The *CF* algorithm can recommend products based on the attributes of the user or the product itself, which can help users quickly find suitable items for themselves, which is the biggest advantage of the *CF* algorithm. In this study, the *CF* algorithm is applied to the communication path recommendation system of mental health and literature. It can recommend appropriate communication paths according to the related attributes and characteristics of mental health and literature, which is similar to the recommendation principle in the field of e-commerce. Figure 2 shows how the *CF* algorithm works. This research mainly adopts an item-based *CF* algorithm. In Figure 2, triangles and rectangles refer to three different items. A, B, C refer to different users. For *CF* algorithm, it mainly includes user-based *CF* algorithm and item-based *CF* algorithm. The user-based *CF* algorithm will recommend items with similar characteristics to the same user, and it also has differences in the item-based algorithm.

This study introduces the process of application of *CF* algorithm in the exploration and application of public health and literature dissemination paths:

(1) The three characteristics of public health and literature dissemination paths will contain huge amounts of data, and this study will be divided into three characteristics for processing. The data between the same features will be calculated for similarity. Equation 1 and Equation 2 show the calculation process of the similarity of the *CF* algorithm, which will calculate the similarity according to the distance between two data.


(1) $$J\left(A,B\right)=\frac{\left|A\cap B\right|}{\left|A\cup B\right|}$$

(2) $$\cos\theta =\frac{a\cdot b}{\left|a\right|\left|b\right|}$$

(2) After the similarity calculation of the two vectors is completed, it needs to further calculate the association similarity between the two vectors. Equation 3 and Equation 4 show the calculation method of the association similarity.


(3) $$sim\left(i,j\right)=\frac{\sum_{u\in U}\left(R_{u,i}-R_{i}\right)\left(R_{u,j}-R_{j}\right)}{\sqrt{\sum_{u\in U}{\left(R_{u,i}-R_{i}\right)}^{2}}\sqrt{\sum_{u\in U}{\left(R_{u,j}-R_{j}\right)}^{2}}}$$

(4) $$sim\left(i,j\right)=\frac{\sum_{u\in U}\left(R_{u,i}-R_{u}\right)\left(R_{u,j}-R_{u}\right)}{\sqrt{\sum_{u\in U}{\left(R_{u,i}-R_{u}\right)}^{2}}\sqrt{\sum_{u\in U}{\left(R_{u,j}-R_{u}\right)}^{2}}}$$

(3) The third step of the *CF* algorithm is to adjust the cosine similarity. This is because the calculation process of cosine similarity does not consider the scores between different users and different items, which easily leads to extreme situations. Equation 5 shows the calculation criterion for adjusting the cosine similarity.


(5) $$\cos\theta =\frac{\sum_{k=1}^{n}x_{1k}x_{2k}}{\sqrt{\sum_{k=1}^{n}x_{1k}{}^{2}}\sqrt{\sum_{k=1}^{n}x_{2k}{}^{2}}}$$

(4) The fourth step of the *CF* algorithm is the calculation process of the weighted average summation. Equation 6 shows the calculation of the weighted average. It is the average processing of the acquisition scores of all user situations.


(6) $$P_{u,i}=\frac{\sum_{all,items}\left(s_{i},N*R_{u},N\right)}{\sum_{all,items}\left(\left|s_{i},N\right|\right)}$$

(5) Equation 7 shows the process of a regression calculation of the *CF* algorithm.


(7) y=ax+b

Application of Atrous Convolution in Mental Health Education.

**Figure 3:**
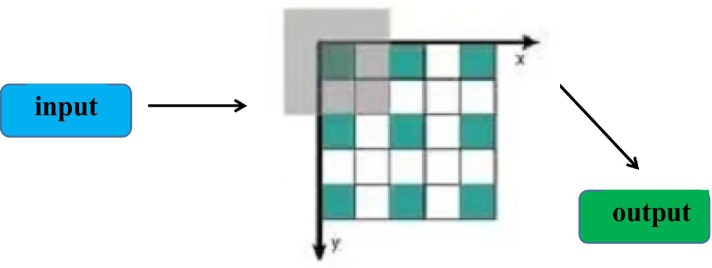
The application of ACNN in the dissemination of mental health literature.

Atrous convolution is a variant of convolutional neural networks, and its filters are processed differently compared to convolutional neural networks. The advantage of atrous convolutional neural network is to deal with large data sets, it will reduce the amount of parameter operation without reducing the accuracy of feature operation. It extracts important features and ignores features with lower weights. Figure 3 shows the workflow of ACNN. Because the features involved in the traditional process of mental health literature are relatively auxiliary, and the computational load of these features is relatively large, which reflects the advantages of ACNN. Both CNN and ACNN can complete the extraction of relevant features and prediction tasks in the process of mental health communication. ACNN has less parameters and requires less running time than CNN methods.

Both CNN and ACNN will involve hyper-parameters, and changes in hyper-parameters will also affect the accuracy of the neural network. Therefore, the training process of ACNN is also a process of continuously adjusting parameters. Equation 8 shows the size calculation method of ACNN output features.


(8) $$S_{out}=\frac{S_{in}+2pading-S_{kenal}}{step}+1$$

Equation 9 shows the calculation method of the input feature size of ACNN, which is different from the calculation method of CNN. Equation 10 shows how the feature size is adjusted.


(8) $$S_{in}=\left(S_{out}-1\right)\times step+S_{kenal}-2pading$$

(9) $$V_{i}=V_{i-1}+S_{kenal-i}\times \overset{i-1}{\underset{i=1}{\prod }}step_{i-1}$$

There are two kinds of functions in any kind of neural network: activation function and loss function. The loss function calculates the error between the predicted and actual values of the characteristics of the mental health literature dissemination process. Most neural networks use a mean squared error loss function, as shown in Equation 11.


(11) $$L=MSE\left(q^{real},q^{pre}\right)=\frac{1}{nm}\overset{N}{\underset{k=1}{\sum }}\overset{M}{\underset{j=1}{\sum }}{\left(q_{kj}{}^{real}-q_{kj}{}^{pre}\right)}^{2}$$

## Result analysis and discussion

This research mainly explores the application of new media technology and big data technology in the process of mental health and literature communication. Mental health is an important matter, and different groups of people have different views on mental health issues. Mental health also has a specific and important relationship with the humanistic spirit. Literary works also have an important healing effect on mental health. This study uses new media technology to study the path problem in mental health and literary communication. ACNN and *CF* algorithms in big data technology will be used to extract features related to mental health communication and it can also complete the recommendation of mental health communication paths. Big data technology requires a large amount of mental health communication-related data. This study selected a mental health-related dataset of a psychological unit in Shenzhen. Shenzhen has the characteristics of dense population and high living pressure. If the data set is more representative, Shenzhen is selected as the research object.

**Figure 4:**
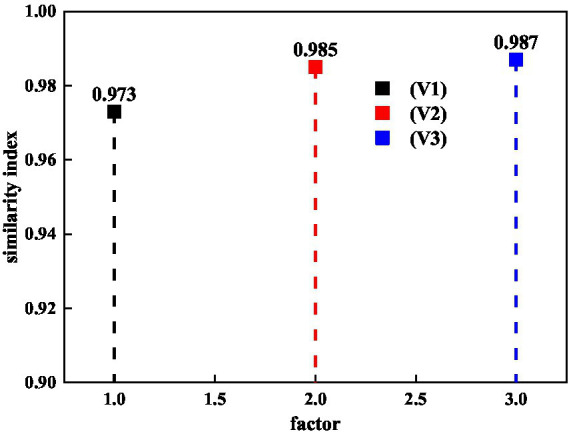
Similarity exponential distributions of mental health and literature transmission paths for *CF* algorithms.

The *CF* algorithm will be used to complete the recommendation of communication paths related to mental health and literature, and it will be based on the mental health-related features extracted by ACNN. The similarity index of the *CF* algorithm is a key evaluation index to measure the success of the recommendation. Figure 4 shows the similarity index between mental health and literature dissemination path recommendations. V1 represents the mental health content characteristics of mental health communication, V2 represents the mental health population preference characteristics, and V3 represents the mental health transmission path characteristics. For the *CF* algorithm, the similarity index reaches 0.9, which shows that the *CF* algorithm has good practical value in the recommendation of mental health and literature. From Figure 4, it can also be seen that the similarity index of the three characteristics recommended by mental health and literature communication paths exceeds 0.973, and the largest similarity index also reaches 0.987. This can already show that the *CF* algorithm is used in the relationship between mental health and literature communication paths. The three features have good application value. The reason why the content features in the mental health communication path is the smallest may be because the content of mental health has relatively large fluctuations or there are fewer data sets of features of mental health content.

**Figure 5:**
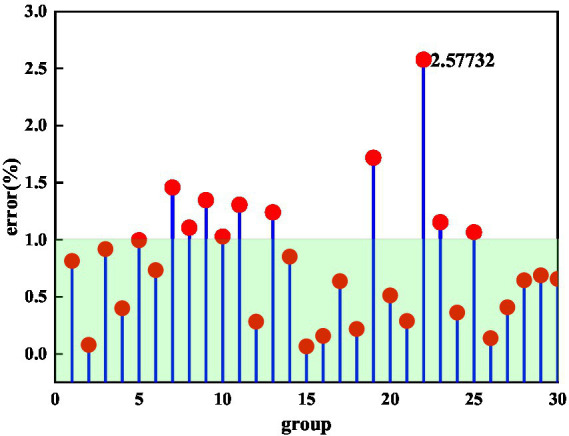
Content feature prediction error of mental health and literature dissemination paths.

The process of mental health and literature dissemination has an important relationship with the content, dissemination path and people’s preferences of mental health dissemination. The relationship between these three mental health characteristics and the transmission path is also relatively complex, and these complex relationships are also difficult to discover by artificial methods. ACNN can be used to establish the relationship between mental health and literary characteristics and transmission paths. Figure 5 demonstrates the prediction error of the content features of mental health and literature using the ACNN method. In Figure 5, the area of the green area represents the characteristics of the communication content in the process of mental health communication within 1% of the error range. This study selected 30 sets of data to illustrate the error of ACNN in predicting the characteristics of mental health content. Overall, the prediction errors of the 30 groups of mental health content characteristics were all within a reasonable range. And most of the errors are within 2%. The prediction error for only one set of content features exceeds 2.5%. This also shows that the ACNN method is suitable for predicting the content characteristics of mental health and literature. It can be seen from Figure 5 that the prediction error of the mental health content features has a relatively large fluctuation, which may be due to the wide distribution of the mental health-related content dataset, which is also beneficial to the ACNN method.

**Figure 6:**
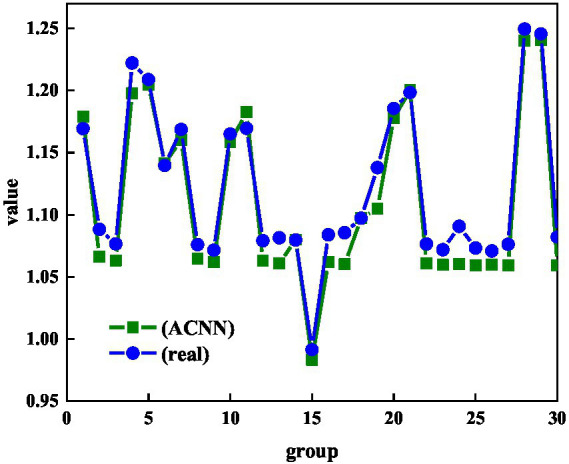
Distribution of predicted and actual values of communication medium characteristics of mental health and literature.

In today’s era, there are many types of new media technology media. Different groups of people have different needs for the communication medium of mental health. Appropriate mental health communication media is also the key to success. This study also separately analyzes the accuracy of ACNN in predicting the transmission medium of mental health and literature. It also digitizes the propagation medium, which enables quantitative analysis of the propagation medium. Figure 6 shows the distribution of predicted values for the media characteristics of mental health and literature. It can be seen from Figure 6 that there are large fluctuations in the 30 groups of different propagation media, and there are also many wave peaks and wave troughs. However, the ACNN method can also effectively and accurately predict the characteristics of the communication medium of mental health and literature. The reason for the large fluctuations in the characteristics of the communication medium in the process of mental health communication is that there are large differences in the characteristics of different groups for the test set. Whether it is the peaks or troughs of the characteristics of the mental health transmission medium, ACNN will compare the predictions of the eigenvalues of the transmission medium.

**Figure 7:**
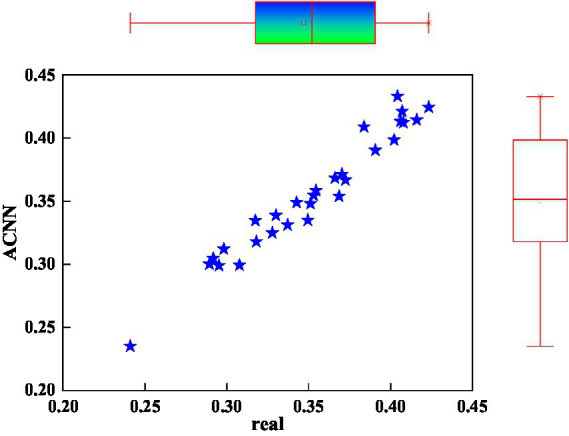
Predicted value distributions of people’s preferences in mental health and literature.

People’s preferences are also an important factor affecting mental health and the path of literature dissemination. Different people have different preferences for new media platforms or new media. This requires different media and different new media platforms to distribute different content of mental health and literature according to people’s preferences. Figure 7 shows the predicted distribution of people’s preference traits for mental health and literary communication using the ACNN method. In Figure 7, the upper box represents the box distribution of the predicted value of the psychologically healthy crowd preference feature. The boxes on the right represent the distribution of actual values of the mental health traditions of population preference characteristics. First of all, from the perspective of linear correlation, the data of the 30 groups of people’s preference eigenvalues are well distributed near the linear function, and the linear correlation coefficient has reached a high value. Then, from the box plot of the crowd preference characteristics, the predicted box plot of the crowd preference characteristics of mental health and literature communication is in good agreement with the actual value of the box plot, whether it is the distribution of values or the shape of the box. The data points of people’s preference feature of mental health communication are distributed on both sides of the linear function, which shows that people’s preference features have a specific and better linear correlation. This refers to the distribution of blue dots. This shows that the ACNN method can also better predict the population preference characteristics related to mental health and literature dissemination paths.

**Figure 8:**
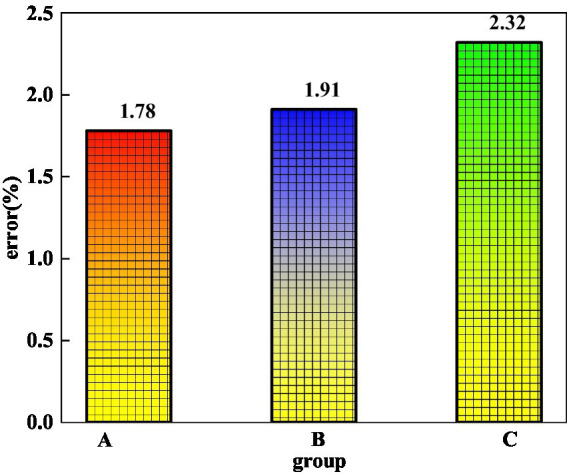
Prediction errors of three characteristics related to mental health and literature dissemination paths.

The aforementioned studies have analyzed the predicted distributions of three traits related to mental health and literary dissemination paths. The average error can reflect the overall prediction situation, which is an analysis of the overall prediction of mental health and literature communication characteristics. Figure 8 shows the average prediction error of three traits related to mental health and literature’s dissemination path. On the whole, the prediction errors of the three characteristics related to mental health and literature dissemination paths are relatively small, and the largest prediction error is only 2.32%. This part of the error is derived from the prediction error of the preference characteristics of the crowd, which is mainly due to the characteristics of the crowd. There are more differences in preferences for different groups of people, and this part of the characteristics is also more related to the content of mental health. The smallest error is only 1.78%. For the error of the mental health transmission path, the prediction error is only 1.91%, which is also a relatively small error.

## Conclusion

With the continuous progress of life and the rapid development of the economy, people’s pressure is increasing, which will bring about greater mental health problems. However, mental health issues are easily overlooked by most people. The dissemination and advocacy of mental health issues is particularly important. Mental health problems also have a specific and important relationship with the continuous lack of humanistic spirit. People’s continuous pursuit of psychological privacy and certain problems in the transmission path of mental health problems are also factors that lead to the failure of mental health knowledge publicity. Mental health is a manifestation of the lack of humanistic spirit, and the dissemination of literary works is conducive to alleviating mental health problems. The failure to spread mental health problems can easily lead to a wider range of mental health problems. With the development of new media technology and more communication media, this has brought new opportunities for the publicity and development of mental health issues.

This research uses new media technology and big data technology to study the problem of mental health and the communication path of literature. The ACNN method in big data technology mainly extracts predictions of features related to mental health dissemination, and the *CF* algorithm recommends the dissemination path of mental health knowledge. First, this study analyzes the performance of *CF* algorithm in mental health and literature recommendation. It can be seen that the maximum similarity index of *CF* algorithm reaches 0.987, and the minimum similarity index also reaches 0.973. This shows that the *CF* algorithm can better recommend new media for mental health and literature based on the characteristics of mental health transmission paths. Then, it analyzes the accuracy of the ACNN method in predicting the characteristics of transmission paths for mental health and literature. It can also be seen from the research that the largest prediction error is only 2.32% for the characteristics of mental health and literature dissemination paths. This study can address mental health issues and the effectiveness of literary dissemination in real life. It can make more people aware of mental health issues under the premise of protecting privacy.

## Data availability statement

The original contributions presented in the study are included in the article/supplementary material, further inquiries can be directed to the corresponding author.

## Author contributions

The author confirms being the sole contributor of this work and has approved it for publication.

## Funding

Ministry of Education of the People’s Republic China: Research on the Relations between Literature, art and Modern Media (13JJD750010).

## Conflict of interest

The author declares that the research was conducted in the absence of any commercial or financial relationships that could be construed as a potential conflict of interest.

## Publisher’s note

All claims expressed in this article are solely those of the authors and do not necessarily represent those of their affiliated organizations, or those of the publisher, the editors and the reviewers. Any product that may be evaluated in this article, or claim that may be made by its manufacturer, is not guaranteed or endorsed by the publisher.
